# Are standard doses of piperacillin sufficient for critically ill patients with augmented creatinine clearance?

**DOI:** 10.1186/s13054-015-0750-y

**Published:** 2015-01-30

**Authors:** Andrew A Udy, Jeffrey Lipman, Paul Jarrett, Kerenaftali Klein, Steven C Wallis, Kashyap Patel, Carl MJ Kirkpatrick, Peter S Kruger, David L Paterson, Michael S Roberts, Jason A Roberts

**Affiliations:** Department of Intensive Care and Hyperbaric Medicine, The Alfred Hospital, Commercial Road, Melbourne, Victoria 3181 Australia; Burns, Trauma, and Critical Care Research Centre, The University of Queensland, Butterfield Street, Brisbane, Queensland 4029 Australia; Department of Intensive Care Medicine, Royal Brisbane and Women’s Hospital, Butterfield Street, Brisbane, Queensland 4029 Australia; Statistics Unit, QIMR Berghofer Medical Research Institute, Herston Road, Brisbane, Queensland 4029 Australia; Faculty of Pharmacy and Pharmaceutical Sciences, Monash University, Royal Parade, Melbourne, Victoria 3052 Australia; Department of Intensive Care Medicine, Princess Alexandra Hospital, Ipswich Road, Brisbane, Queensland 4102 Australia; Department of Infectious Diseases, Royal Brisbane and Women’s Hospital, Butterfield Street, Brisbane, Queensland Australia; Centre for Clinical Research, The University of Queensland, Butterfield Street Brisbane, Queensland 4029, Australia; School of Pharmacy and Medical Sciences, University of South Australia, North Terrace, Adelaide, South Australia 5000 Australia

## Abstract

**Introduction:**

The aim of this study was to explore the impact of augmented creatinine clearance and differing minimum inhibitory concentrations (MIC) on piperacillin pharmacokinetic/pharmacodynamic (PK/PD) target attainment (time above MIC (*f*T_>MIC_)) in critically ill patients with sepsis receiving intermittent dosing.

**Methods:**

To be eligible for enrolment, critically ill patients with sepsis had to be receiving piperacillin-tazobactam 4.5 g intravenously (IV) by intermittent infusion every 6 hours for presumed or confirmed nosocomial infection without significant renal impairment (defined by a plasma creatinine concentration greater than 171 μmol/L or the need for renal replacement therapy). Over a single dosing interval, blood samples were drawn to determine unbound plasma piperacillin concentrations. Renal function was assessed by measuring creatinine clearance (CL_CR_). A population PK model was constructed, and the probability of target attainment (PTA) for 50% and 100% *f*T_>MIC_ was calculated for varying MIC and CL_CR_ values.

**Results:**

In total, 48 patients provided data. Increasing CL_CR_ values were associated with lower trough plasma piperacillin concentrations (*P* < 0.01), such that with an MIC of 16 mg/L, 100% *f*T_>MIC_ would be achieved in only one-third (*n* = 16) of patients. Mean piperacillin clearance was approximately 1.5-fold higher than in healthy volunteers and correlated with CL_CR_ (*r* = 0.58, *P* < 0.01). A reduced PTA for all MIC values, when targeting either 50% or 100% *f*T_>MIC_, was noted with increasing CL_CR_ measures.

**Conclusions:**

Standard intermittent piperacillin-tazobactam dosing is unlikely to achieve optimal piperacillin exposures in a significant proportion of critically ill patients with sepsis, owing to elevated drug clearance. These data suggest that CL_CR_ can be employed as a useful tool to determine whether piperacillin PK/PD target attainment is likely with a range of MIC values.

## Introduction

Effective antibacterial therapy is crucial for improving outcomes for patients with sepsis [1]. Current international guidelines stress the importance of early administration of broad-spectrum agents [2], with the caveat that significant dose adjustment may be required in patients who are critically ill. This is a reflection of the unique physiology often encountered in these patients [3], which may dramatically distort pharmacokinetics (PK). Changes in antibacterial volume of distribution, plasma protein binding and drug clearance are well described in the literature [4]. They lead to poorly predictable and often subtherapeutic plasma concentrations [5].

The minimum antibacterial exposure required in clinical practice to maximize bacterial killing and optimize clinical outcomes remains controversial. For β-lactams, preclinical studies support maintaining prolonged free drug concentrations above the minimum inhibitory concentration (MIC) of the likely pathogen [6]. *In vivo* animal data indicate that this period should be at least 40% to 70% of the dosing interval (40% to 70% *f*T_>MIC_) [7], although various retrospective clinical evaluations have recommended more aggressive targets, such as 100% *f*T_>MIC_ [8] and trough concentration to MIC ratios (C_min_:MIC) >5 [9]. More recently, in a large multinational point prevalence study of antibacterial concentrations in critical illness, researchers demonstrated that clinical failure is three times more likely when β-lactam exposure is less than 50% *f*T_>MIC_ [5] and that increasing exposures are associated with an increased likelihood of clinical cure.

Whereas these data demonstrate why achievement of target drug exposures is important, they do not robustly define which patients are at risk of subtherapeutic dosing. Clinicians commonly consider the likely pathogen, its susceptibility profile and the potential for drug toxicity when selecting an empirical dosing regimen. An assessment of renal function is common, with evidence of acute or chronic renal impairment often triggering dose reduction to avoid drug accumulation. For β-lactams, the converse—dose escalation in the setting of augmented renal function—has been infrequently reported in clinical practice.

Previously published data, however, suggest that patients with augmented renal clearance (ARC), which often manifests as an elevated urinary creatinine clearance (CL_CR_), are at particular risk of subtherapeutic β-lactam concentrations [10,11]. This, therefore, represents an attractive measure to guide dose selection, although few data are available that integrate CL_CR_, pathogen susceptibility and drug exposures. As such, the aim of this analysis was to explore the impact of elevated CL_CR_ and different pathogen susceptibilities, on piperacillin pharmacokinetic/pharmacodynamic (PK/PD) target attainment in critically ill patients receiving intermittent dosing.

## Material and methods

### Setting

This single-centre observational study was undertaken in a tertiary level, university-affiliated intensive care unit (ICU). Ethical approval was obtained from the Royal Brisbane and Women’s Hospital Human Research Ethics Committee (HREC 2007/188), with written informed consent obtained from either the patient or the patient’s nominated substitute decision-maker.

### Study population

Patients were eligible for enrolment if they were between 18 and 80 years of age and were receiving piperacillin-tazobactam for the treatment of sepsis (defined as presumed or confirmed nosocomial infection while manifesting a systemic inflammatory response syndrome [12]). Patients were excluded if they (1) did not have an intraarterial line inserted as part of routine management (to allow repeated plasma sampling without additional venipuncture), (2) had renal impairment (defined by a plasma creatinine concentration (CR) greater than 171 μmol/L or the need for renal replacement therapy) or (3) had a history of allergy to piperacillin or iodine. This, therefore, represents a convenience sample of critically ill septic patients admitted to our institution without significant renal impairment.

### Study protocol

The protocol pertaining to this study has been published in detail elsewhere [13]. In brief, 4.5 g of piperacillin-tazobactam (Tazocin EF; Pfizer Australia Pty Ltd, West Ryde, NSW, Australia) diluted in 50 ml of 0.9% sodium chloride was administered over 20 minutes as part of the patient’s prescribed course of therapy. Blood samples to determine plasma piperacillin concentrations were drawn predose and at 20 minutes (end of infusion), 40 minutes, 60 minutes, 210 minutes and 360 minutes. All urine samples were collected via an indwelling urinary catheter over the dosing interval, following which urine volume and urinary CR concentration were determined by laboratory analysis. Plasma CR concentrations on the day of investigation were used to calculate CL_CR_. No specific CL_CR_ value was used to define renal impairment or ARC, as our aim was to use these measures as continuous variables in analysis.

Additional data, including the requirement for mechanical ventilation, vasopressor support, modified Sequential Organ Failure Assessment (SOFA) score (excluding the neurological component) and 24-hour fluid balance, were documented on the day of drug administration. Admission Acute Physiology and Chronic Health Evaluation (APACHE) II score, body mass index (BMI) and ICU and in-hospital mortality were also recorded.

### Sample handling, storage and measurement

Blood samples were immediately placed on ice and centrifuged within 60 minutes at 3,000 rpm for 10 minutes. Plasma samples were then stored at −80°C until analysis. An ultraviolet high-performance liquid chromatography (HPLC-UV) assay was used to measure piperacillin concentrations in plasma. All bioanalytical techniques were validated and conducted in accordance with the criteria of the US Food and Drug Administration’s guidance for industry on bioanalysis [14]. To isolate the unbound fraction for analysis, protein-bound piperacillin was removed from the plasma sample with centrifugal filter devices (Centrifree 30,000 NMWL; Merck Millipore, Tullagreen, Ireland). The lower limit of quantification for unbound piperacillin concentrations was 1 mg/L.

### Plasma concentration data

The final (360 minutes) plasma sample was taken as the trough unbound piperacillin concentration (C_min_). These samples were then compared to an MIC of 16 mg/L to determine whether 100% *f*T_>MIC_ would have been achieved. This represents the highest MIC for susceptible bacteria (for example, the *Pseudomonas aeruginosa* MIC is 16 mg/L for piperacillin/tazobactam) as per the European Committee on Antimicrobial Susceptibility Testing (EUCAST) [15]. We chose to assess the adequacy of dosing in terms of this ‘worst-case scenario’, as MIC data are often not initially available to the clinician. The 100% *f*T_>MIC_ target was utilized in this analysis, as more aggressive drug exposures have been demonstrated to improve both microbiological and clinical outcomes [5,8,9].

### Population pharmacokinetics modelling

Concentration-time data were analysed using nonlinear mixed-effects modelling (NONMEM version 7.1; GloboMax, Ellicott City, MD, USA). A Digital Fortran compiler was used, and the runs were executed using Wings for NONMEM [16]. The first-order conditional estimation method with interaction was used throughout model building. Unbound plasma piperacillin concentrations were fitted to one-, two- or three-compartment linear models using subroutines from the NONMEM library. Between-subject variability (BSV) was evaluated using an exponential variability model. Various models for residual unexplained variability (RUV) were also tested.

### Pharmacokinetics model diagnostics

Visual inspection of diagnostic scatter plots and the NONMEM objective function value (OFV) were used to evaluate goodness of fit. Statistical comparison of nested models was undertaken in the NONMEM program using log-likelihood ratios, which are assumed to be χ^2^-distributed. On the basis of a χ^2^ test of the difference in OFV, a decrease in the OFV of 3.84 units (*P* < 0.05) for 1 degree of freedom was considered statistically significant. Decreases in BSV of one of the parameters of at least 10% were also accepted for inclusion in a more complicated model.

### Population pharmacokinetics bootstrap

A nonparametric bootstrap method (*n* = 1,000) was used to study the uncertainty of the pharmacokinetic parameter estimates in the final model. From the bootstrap empirical posterior distribution, we were able to obtain the 95% confidence interval (2.5% to 97.5% percentiles) for the parameters, as described previously [17].

### Probability of target attainment

Monte Carlo simulations performed in NONMEM were employed to determine the probability of target attainment (PTA) with varying MIC (2 to 64 mg/L) and CL_CR_ (10 to 300 ml/min in 10-ml/min increments) values using a standard 4.5-g piperacillin-tazobactam dose administered every 6 hours as an intermittent 20-minute infusion. The PTAs for achieving 50% *f*T_>MIC_ and 100% *f*T_>MIC_ with piperacillin were calculated.

### Cumulative fraction of response

The cumulative fraction of response (CFR) determines the likely success of treatment by comparing the pharmacodynamic exposure (PTA) against the MIC distribution of a specific population of microorganisms [18]. The wild-type MIC distribution for *Pseudomonas aeruginosa* against piperacillin was obtained from the EUCAST database [15]. The CFR was calculated for both 50% *f*T_>MIC_ and 100% *f*T_>MIC_ by using a range of CL_CR_ values. Dosing was *a priori* considered successful if the CFR was at least 80%.

### Statistical analysis

Continuous data are presented as the mean (standard deviation) or median (interquartile range). Categorical data are presented as counts (%). Correlation was assessed by means of a scatter graph and Pearson correlation coefficient (*r*). For comparisons between groups, we used an independent Student’s *t*-test, Mann–Whitney *U* test, or one-way analysis of variance for continuous data, as well as a χ^2^ or Fisher’s exact test for categorical data, where analysis assumptions were met. A *P*-value <0.05 was considered statistically significant, and all analyses were performed using SPSS version 21 software (IBM SPSS, Chicago, IL, USA).

## Results

### Demographic data

Forty-eight patients were included in the analysis. All patients were receiving 4.5 g of piperacillin-tazobactam by intermittent IV infusion over 20 minutes every 6 hours for treatment of presumed or confirmed nosocomial infection. Demographic, admission, anthropometric, illness severity and outcome data are presented in Table 1. Sampling occurred after a median of 9 (4 to 13) doses and, on average, 5.2 (3.7) days postadmission to the ICU. Half of the patients were receiving piperacillin-tazobactam for treatment of nosocomial pneumonia. The cohort was relatively young and overweight with moderate to severe illness severity. The vast majority required invasive mechanical ventilation (>90%), whereas only one-fourth needed vasopressor support. As per the inclusion criteria, plasma CR concentrations were relatively low (79.5 (31.4) μmol/L). The mean CL_CR_ over the study period was 122 (59.2) ml/min, although there was significant variability between patients: 15 patients had a CL_CR_ <90 ml/min, 22 had a CL_CR_ >130 ml/min and 16 had a CL_CR_ >150 ml/min.

**Table 1:** **Demographic, anthropometric, illness severity and outcome data** ^**a**^

**Variable**			***N*** **= 48**
Age, yr			47.3 (17.9)
Male sex, *n* (%)			27 (56.3)
Height, m			1.70 (0.11)
Weight, kg			88.4 (24.2)
BMI, kg/m^2^			30.7 (8.78)
BSA, m^2^			1.98 (0.26)
Admission category/system, *n* (%)			
	Medical		*n* = 13
		Cardiac	1 (7.7)
		Gastrointestinal	3 (23.1)
		Neurological	2 (15.4)
		Respiratory	5 (38.5)
		Primary bacteraemia	1 (7.7)
		Oncology	1 (7.7)
	Surgical		*n* = 13
		Gastrointestinal	7 (53.8)
		Gynaecological	1 (7.7)
		Maxillofacial	2 (15.4)
		Neurological	2 (15.4)
		Vascular	1 (7.7)
	Trauma		*n* = 22
		Burns	6 (27.3)
		Facial	1 (4.5)
		Abdominal	4 (18.2)
		Neurological	5 (22.7)
		Orthopaedic	3 (13.6)
		Thoracic	3 (13.6)
APACHE II score			19.4 (6.79)
Modified SOFA score, median (IQR)			3.5 (2 to 6)
Mechanical ventilation, *n* (%)			45 (93.8)
Use of vasopressors, *n* (%)			12 (25.0)
Presumed/confirmed site of infection, *n* (%)			
	Intraabdominal		14 (29.2)
	Skin/soft tissue		5 (10.4)
	Respiratory		24 (50.0)
	Urinary		1 (2.1)
	Primary bacteraemia		1 (2.1)
	Unknown		3 (6.3)
24-hr fluid balance, ml			553 (1836)
Plasma CR, μmol/L			79.5 (31.4)
Measured CL_CR_, ml/min			122 (59.2)
ICU length of stay, days			18.2 (11.5)
ICU mortality, *n* (%)			4 (8.3)
Hospital mortality, *n* (%)			5 (10.4)

^a^APACHE, Acute Physiology and Chronic Health Evaluation; BMI, Body mass index; BSA, Body surface area; CL_CR_, Creatinine clearance; CR, Creatinine; ICU, Intensive care unit; IQR, Interquartile range; SD, Standard deviation; SOFA, Sequential Organ Failure Assessment. Data presented are mean (SD) unless otherwise stated.

### Plasma concentration data

**Figure 1:**
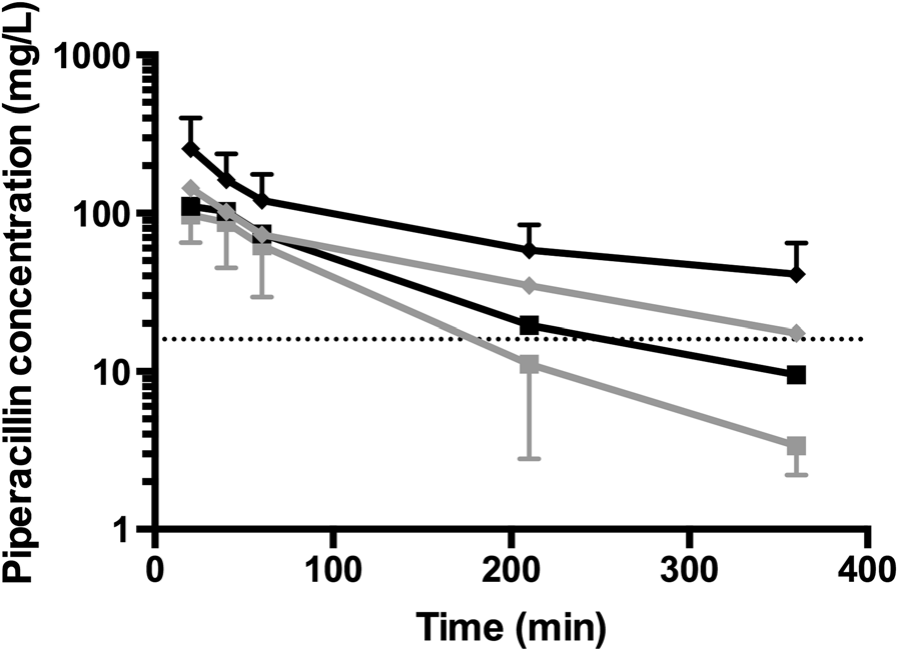
**Piperacillin concentrations over time grouped according to creatinine clearance quartile.** Mean piperacillin concentration (log_10_ scale) and time grouped according to creatinine clearance (CL_CR_) quartile: <68 ml/min (black diamonds), 68 to 114 ml/min (grey diamonds), 115 to 170 ml/min (black squares) and >170 ml/min (grey squares). The dotted line at 16 mg/L represents minimum inhibitory concentration.

In 11 patients, the C_min_ value was either below the lower limit of detection (*n* = 10) or not available (*n* = 1). Using an MIC of 16 mg/L, piperacillin intermittent infusion would achieve 100% *f*T_>MIC_ (C_min_ >16 mg/L) in only 16 (34.0%) of 47 patients. Older age (*P* = 0.01), higher modified SOFA score (*P* < 0.01), lower CL_CR_ (*P* < 0.01), greater positive 24-hour fluid balance (*P* = 0.04) and more frequent application of vasopressor therapy (*P* = 0.01) were characteristics of patients likely to have higher C_min_ values, and therefore achieve this PK/PD target. No statistically significant differences were noted in BMI, APACHE II scores or the need for mechanical ventilation. Unbound plasma piperacillin concentration time profiles grouped by CL_CR_ quartile are presented in Figure 1. Higher CL_CR_ was significantly associated with lower unbound trough plasma piperacillin concentration (*P* < 0.01).

### Pharmacokinetic model building

The time course of unbound plasma piperacillin concentrations was best described by a two-compartment linear model with combined residual error and BSV on the volume of the central (V_c_) and peripheral compartments (V_p_), drug clearance (CL) and an infusion lag descriptor (ALAG; defined as the time taken for the residual drug to get through the IV line after completion of the 20-minute infusion, which was included as zero order input). The only covariate that improved the fit of the model was, for piperacillin CL, CL_CR_ normalized to 100 ml/min, which decreased the OFV by 18.62 (*P* < 0.01). In the final model, this was described as follows:

$$ \mathrm{TVCL} = \mathrm{C}\mathrm{L} \times {\mathrm{CL}}_{\mathrm{CR}}/100, $$

where TVCL is the typical value of piperacillin CL. The mean parameter estimates from the final covariate model, as well as the 95% confidence intervals from all bootstrap runs, are shown in Table 2. Total volume of distribution (V_d_) was calculated as the sum of V_c_ and V_p_. The goodness-of-fit plots were acceptable with an *r*-value of 0.95 for the observed versus individual predicted concentrations.

**Table 2:** **Mean parameter estimates and bootstrap mean (95% confidence interval) estimates for the final population pharmacokinetic model** ^**a**^

**Parameter**		**Model**	**Bootstrap**		
		**Mean**	**Mean**	**95% confidence interval**	
				**2.5%**	**97.5%**
Fixed effects					
	CL (L/hr)	16.3	16.2	14.0	18.9
	V_c_ (L)	19.9	15.9	0.7	27.8
	V_p_ (L)	18.8	21.3	11.1	32.3
	Q (L/h)	37.3	42.9	22.0	73.3
	ALAG (hr)	0.8	0.13	0.01	0.31
Random effects BSV (% CV)					
	CL (L/hr)	56.0	55.3	45.2	65.4
	V_c_ (L)	29.6	23.5	0.2	45.7
	V_p_ (L)	67.6	69.2	24.6	158.4
	ALAG (hr)	0.3	0.4	0.05	0.8
Random error					
	RUV (% CV)	1.0	1.3	0.3	3.1
	RUV (SD, mg/L)	0.3	0.3	0.2	0.4

^a^ALAG, Infusion lag time; BSV, Between-subject variability; CL, Clearance; CV, Coefficient of variation; Q, Intercompartmental clearance; RUV, Residual unexplained variability; SD, Standard deviation; V_c_, Volume of the central compartment; V_p_, Volume of the peripheral compartment.

**Figure 2:**
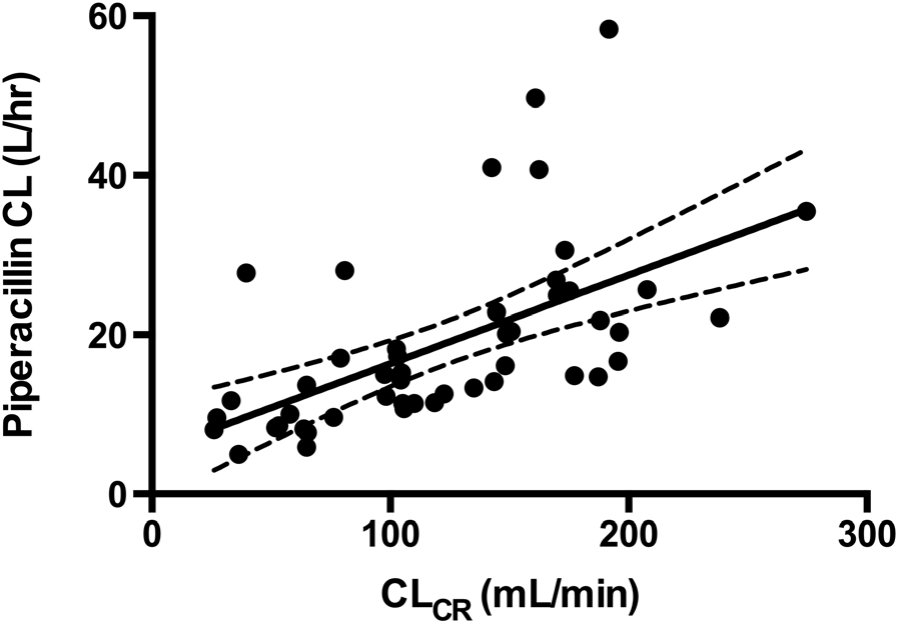
**Piperacillin drug clearance compared with creatinine clearance.** Scatter plot of piperacillin clearance (CL) and creatinine clearance (CL_CR_). A linear regression line (solid) with 95% confidence interval (dashed line) has been fitted to the data points (*r* = 0.58, *P* < 0.001).

**Figure 3:**
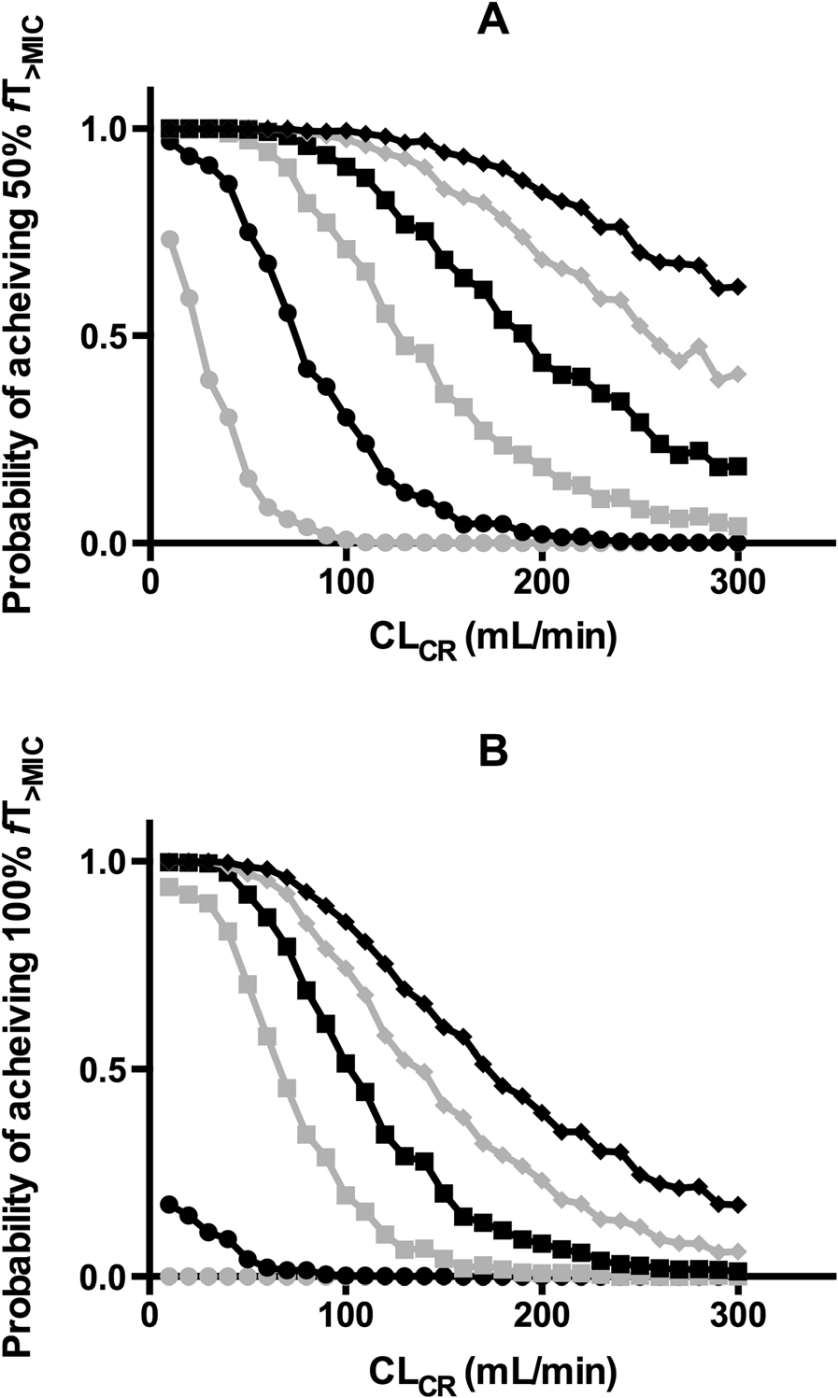
**Probability of target attainment with varying creatinine clearance.** Graphs depict the probability (%) of attaining 50% **(A)** and 100% **(B)** time above the minimum inhibitory concentration (fT_>MIC_) following a 4.5 g piperacillin-tazobactam dose administered every 6 hours as an intermittent 20-minute infusion, and CLCR values. Varying minimum inhibitory concentrations (MIC) are displayed: 2 mg/L (black diamonds), 4 mg/L (grey diamonds), 8 mg/L (black squares), 16 mg/L (grey squares), 32 mg/L (black circles), and 64 mg/L (grey circles).

Those patients who would fail to achieve 100% *f*T_>MIC_ (16 mg/L) had higher drug CL (*P* < 0.01) and no statistically significant difference in piperacillin V_d_ (*P* = 0.647). As demonstrated in Figure 2, a moderate correlation was noted between CL_CR_ and piperacillin CL overall (*r* = 0.58, *P* < 0.01). The PTAs for 50% *f*T_>MIC_ and 100% *f*T_>MIC_ with varying MIC and CL_CR_ values are presented in Figure 3.

The CFR for piperacillin over a range of CL_CR_ values using the EUCAST MIC distribution for *Pseudomonas aeruginosa* is presented in Table 3.

**Table 3:** **Cumulative fraction of response for piperacillin over a range of creatinine clearance values** ^**a**^

**CL**_**CR**_ **(ml/min)**	**CFR, 50%** ***f*** **T**_**>MIC**_ **(%)**	**CFR, 100% fT**_**>MIC**_ **(%)**
10	**87.2**	**81.3**
30	**86.0**	**80.6**
60	**83.5**	72.6
90	78.6	57.2
120	71.7	40.4
150	62.7	28.5
180	55.5	20.0
210	46.3	13.3
240	40.8	9.9
270	31.0	6.3
300	28.3	4.9

^a^50% *f*T_>MIC_, 50% time above the minimum inhibitory concentration; 100% *f*T_>MIC_, 100% time above the minimum inhibitory concentration; CFR, Cumulative fraction of response; CL_CR_, Creatinine clearance. Data represent the CFR when targeting 50% fT_>MIC_ and 100% fT_>MIC_ with a range of CLCR values following a 4.5 g piperacillin-tazobactam dose administered every 6 hours as an intermittent 20 minute infusion. The wild-type MIC distrubiton of *Pseudomonas aeruginosa* has been employed. Boldface represents those CLCR values where target exposures would be achieved in at least 80% of isolates.

## Discussion

In this clinical analysis, we sought to explore the impact of elevated CL_CR_ and different susceptibilities of bacteria on piperacillin PK/PD target attainment when administered by intermittent infusion in a large cohort of critically ill patients with sepsis. With a MIC at the upper limit of the susceptible range for piperacillin (16 mg/L), only about one-third of patients would achieve 100% *f*T_>MIC_. The primary mechanism underlying this observation appears to be significantly elevated drug CL in parallel with higher CL_CR_ in some patients. This phenomenon of augmented renal clearance [19,20] is being described in critically ill patients with increasing frequency [21,22], which suggests that clinicians should be wary of conventional dosing in this setting. Indeed, the CFR suggests that only with lower CL_CR_ values (<90 ml/min) will an adequate fraction of *Pseudomonas aeruginosa* isolates be suitably covered with this dosing regimen.

Pharmacokinetic modelling for the study cohort revealed a mean piperacillin CL of approximately 1.5-fold that reported in healthy volunteers [23]. Similar derangement in piperacillin CL has been reported in previous studies of critically ill patients [24,25], highlighting the unique PK observed in this setting [4]. Of note, piperacillin V_d_ was also significantly larger overall. Changes in V_d_ have been observed with other β-lactams in critical illness [26] and likely reflect the substantial fluid shifts frequently encountered [27]. Use of unbound piperacillin concentrations in the PK model may also have contributed to the higher V_d_ observed, although use of free concentrations is essential to describe the pharmacologically active fraction of piperacillin.

The specific mechanisms underlying ARC in critically ill patients remain uncertain. Researchers have recently identified a modest correlation between cardiac index and CL_CR_ in patients with sepsis [28], suggesting that increased renal perfusion and solute delivery may be a key factor. Younger age and lower illness severity scores have previously been identified as risk factors for ARC [28,29], which is further demonstrated by our study data. Work by Shimamoto *et al*. highlights the role of systemic inflammation, with an increasing number of systemic inflammatory response syndrome criteria being strongly correlated with higher drug clearance and lower plasma concentrations in nonventilated patients receiving vancomycin [30]. Together, these findings imply an important interaction between systemic inflammation and available organ reserve, factors that are often neglected in antibacterial dosing regimens [31]. As such, the characteristics of patients included in this study (younger age, admission posttrauma and less organ dysfunction) are useful in defining scenarios where a measured CL_CR_ or therapeutic drug monitoring should be employed to determine the need for higher than standard piperacillin dosing.

The prevalence of ARC varies significantly, depending upon the definition employed and institutional case mix [32-35]. In a recent multicentre study of CL_CR_ in critically ill patients with ‘normal’ plasma CR concentrations, researchers identified an incidence of ARC (CL_CR_ ≥130 ml/min/1.73 m^2^) of approximately 65% in the first 7 days in the ICU [36]. Importantly, patients who manifested ARC on day 1 were significantly more likely to do so on subsequent measures, indicating that augmented drug CL is unlikely to be short-lived. A lower incidence (about 52%) of ARC was observed in a mixed cohort of critically ill patients receiving antibacterial therapy, although an association with greater therapeutic failure was demonstrated [29].

Other investigators have highlighted the importance of renal function in informing β-lactam dosing. Patel and colleagues examined piperacillin PK data from 105 hospitalized patients to determine optimal intermittent and extended-infusion dosing in those with renal impairment [37]. Of interest, with an estimated CL_CR_ of 100 ml/min and a traditional regimen of 4.5 g administered IV every 6 hours, the probability of target attainment (50% *f*T_>MIC_) decreased sharply (<80%) with MIC values greater than 4 mg/L [34]. With our data, we identified higher CL_CR_ thresholds (Figure 3), although this likely reflects differences in case mix and the use of measured rather than estimated CL_CR_. Importantly, previous data reinforce the significant disparity between calculated and measured CL_CR_ in patients manifesting ARC [38,39].

More recently, Carlier *et al*. examined meropenem and piperacillin-tazobactam target attainment in a cohort of critically ill patients receiving extended-infusion dosing. In those patients manifesting ARC (CL_CR_ ≥130 ml/min), the probability of achieving either 50% *f*T_>MIC_ or 100% *f*T_>MIC_ was significantly reduced [40]. Regression analysis also identified CL_CR_ as the only significant predictor of target attainment, a finding also demonstrated by Hites *et al*. in their work exploring β-lactam exposure in obese, non-critically ill patients [41]. Our data compare favourably with these studies, although the current analysis involves a wider range of MIC values, highlighting the impact of variable bacterial susceptibility. Pea and colleagues similarly examined meropenem continuous infusion dosing in critically ill patients with severe Gram-negative infection [42]. Dosing nomograms to achieve specific steady-state concentrations were developed with a linear relationship between dose and estimated CL_CR_ [42].

Changing bacterial susceptibility represents a global challenge in medical practice. Our analyses highlight the importance of the MIC as the denominator in the PK/PD relationship in determining adequate antibacterial dosing. Less susceptible pathogens are more common in the ICU [43], and significant regional variation in resistance patterns also exists [44]. In two large antibacterial susceptibility prevalence studies, MIC_90_ values for piperacillin-tazobactam against *Pseudomonas aeruginosa* were reported at or above 64 mg/L [45,46]. Our data suggest that achieving 50% *f*T_>MIC_ in such a scenario is unlikely with conventional piperacillin intermittent dosing and reinforce the use of a much lower susceptibility breakpoint (16 mg/L) with this dosing strategy. However, even in this circumstance, an elevated CL_CR_ will significantly reduce the probability of target attainment, as demonstrated in Figure 3. In contrast, where a highly susceptible pathogen is identified and piperacillin is used as directed therapy, standard doses may still be sufficient, even with extremely high CL_CR_ values. Importantly, this underlines the significance of such microbiological susceptibility data in accurate clinical decision-making.

Our study has several limitations. The data were drawn from a single centre and as such may not be representative of case mix at other institutions. Our inclusion criteria were designed to select a cohort of patients without significant renal impairment, as effective antibacterial dosing in this group is less likely with standard dosing. As such, ARC is likely to have been more common in our study cohort than in a broader ICU population. However, our relatively high plasma CR cutoff allowed us to explore renal function (as determined by CL_CR_) as a covariate in PK modelling. We did not include tazobactam PK, because it is not active against *Pseudomonas aeruginosa*, although, as it is renally eliminated, increased drug CL is also likely in ARC. We examined the likelihood of target attainment using varying susceptibility levels rather than clinical MIC data. This improves generalizability for empiric dosing, although it limits conclusions about the adequacy of drug exposure in any specific patient. Furthermore, given the small sample size, we are unable to make any firm conclusions about the clinical implications of these analyses.

## Conclusions

Our results indicate that an empiric intermittent infusion of 4.5 g of piperacillin-tazobactam is unlikely to achieve optimal piperacillin exposure in a significant proportion of critically ill patients with sepsis, particularly when targeting less susceptible pathogens. This appears to be driven primarily by an increase in drug CL in some patients, and our data reinforce the value of CL_CR_ in predicting whether optimal exposures are likely. As such, clinicians should be wary of the adequacy of conventional dosing in patients manifesting ARC, and research should now be focused on the use of novel administration strategies in such patients, in addition to correlating changes in antibacterial PK with clinical outcomes.

## Key messages

- Elevated CL_CR_ in septic critically ill patients is associated with higher piperacillin CL, a concept referred to as *augmented renal clearance*, or ARC.
- When employing intermittent administration, this will result in suboptimal plasma concentrations for significant periods of the dosing interval.
- Increasing CL_CR_ reduces the probability of target attainment (*f*T_>MIC_), particularly with more resistant organisms.
- When considering the MIC distribution of *Pseudomonas aeruginosa*, 6-hourly dosing of 4.5 g piperacillin-tazobactam is unlikely to provide sufficient piperacillin exposure when the CL_CR_ is ≥90 ml/min.

## Abbreviations

ALAG: Infusion lag descriptor
APACHE: Acute Physiology And Chronic Health Evaluation
ARC: Augmented renal clearance
BMI: Body mass index
BSV: Between-subject variability
CFR: Cumulative fraction of response
CL: Drug clearance
CL_CR_: Creatinine clearance
C_min_: Trough concentration
CR: Creatinine
CV: Coefficient of variation
EUCAST: European Committee on Antimicrobial Susceptibility Testing
*f*T_>MIC_: Fraction of the dosing interval for which the concentration remains above the minimum inhibitory concentration
HPLC-UV: High-performance liquid chromatography-ultraviolet
ICU: Intensive care unit
IDC: Indwelling urinary catheter
IQR: Interquartile range
LOS: Length of stay
MIC: Minimum inhibitory concentration
NONMEM: Nonlinear mixed-effects modelling
OFV: Objective function value
PD: Pharmacodynamic
PK: Pharmacokinetic
PTA: Probability of target attainment
Q: Intercompartmental clearance
RUV: Residual unexplained variability
SD: Standard deviation
SOFA: Sequential Organ Failure Assessment
TVCL: Typical value of piperacillin clearance
V_c_: Volume of the central compartment
V_d_: Volume of distribution
V_p_: Volume of the peripheral compartment
